# Supplementary material for: CauseMap: fast inference of causality from complex time series
Source: PeerJ. 2015 Mar 5;3:e824. doi: 10.7717/peerj.824 (PMC4359046; doi:10.7717/peerj.824)
Supplement: Supplemental Information [file peerj-03-824-s001.docx]

# Paramecium-Didinium system

Didinium is a free-living unicellular carnivore. Paramecium is its prey. More information about this system, as well as interactive graphs of time series and manifold constructions, can be found at: <http://cyrusmaher.github.io/CauseMap.jl/ParaDidiExample.html#paramecium-and-didinium>

# Sensitivity analysis

CauseMap includes plotting functionality to examine the dependence of the maximal crossmap correlation on parameters for E and tau_p. The Figure S1 demonstrates how this signal changes as we use progressively fewer data points from the original dataset. We note that the patterns observed in these plots are maintained better with increasingly less data when compared to the convergence of the cross map correlation with library size. We speculate that this visualization may be useful for identifying promising signals in short time series, in addition to allowing for improved interpretation of optimal values of E and tau_p.

# Fourier transform analysis

We calculated the characteristic frequencies of the paramecium and didinium time series by performing fourier transform analysis using the rfft function in the python module scipy.
