# Supplementary figures and images for: CauseMap: fast inference of causality from complex time series
Source: PeerJ. 2015 Mar 5;3:e824. doi: 10.7717/peerj.824 (PMC4359046; doi:10.7717/peerj.824)

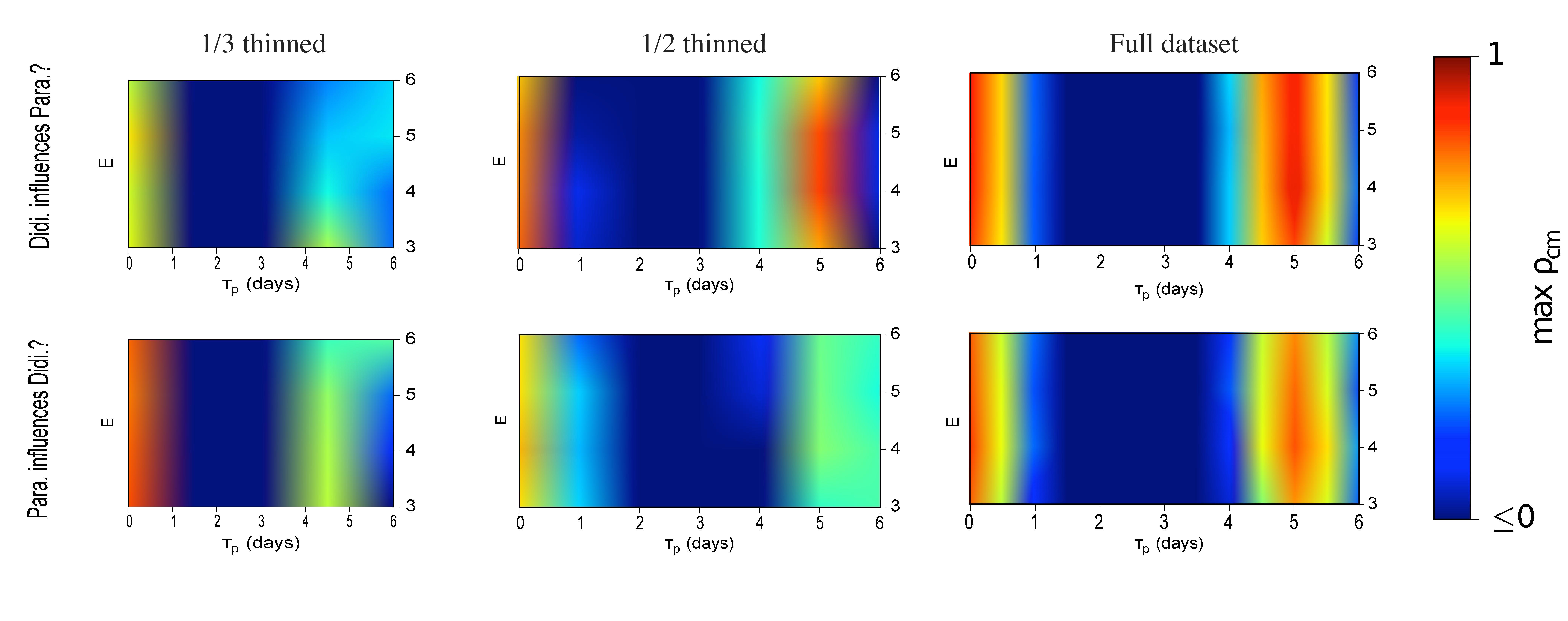

Supplement: Figure S1 [file peerj-03-824-s002.jpg]
